# Superparamagnetic Iron Oxide Nanoparticles for Immunotherapy of Cancers through Macrophages and Magnetic Hyperthermia

**DOI:** 10.3390/pharmaceutics14112388

**Published:** 2022-11-05

**Authors:** Alexandre M. M. Dias, Alan Courteau, Pierre-Simon Bellaye, Evelyne Kohli, Alexandra Oudot, Pierre-Emmanuel Doulain, Camille Petitot, Paul-Michael Walker, Richard Decréau, Bertrand Collin

**Affiliations:** 1 Centre George-François Leclerc, Service de Médecine Nucléaire, Plateforme d’Imagerie et de Radiothérapie Précliniques, 1 rue du Professeur Marion, 21079 Dijon, France; 2 ImViA Laboratory, EA 7535, University of Burgundy, 21000 Dijon, France; 3 UMR INSERM/uB/AGROSUP 1231, Labex LipSTIC, Faculty of Health Sciences, Université de Bourgogne Franche-Comté, 21079 Dijon, France; 4 University Hospital Centre François Mitterrand, 21000 Dijon, France; 5 Synthesis of Nanohybrids (SON) SAS, 21000 Dijon, France; 6 Institut de Chimie Moléculaire de l’Université de Bourgogne, UMR CNRS/uB 6302, Université de Bourgogne Franche-Comté, 21079 Dijon, France

**Keywords:** cancer, immunotherapy, superparamagnetic iron oxide, nanoparticles, macrophages, magnetic hyperthermia, theranostics

## Abstract

Cancer immunotherapy has tremendous promise, but it has yet to be clinically applied in a wider variety of tumor situations. Many therapeutic combinations are envisaged to improve their effectiveness. In this way, strategies capable of inducing immunogenic cell death (e.g., doxorubicin, radiotherapy, hyperthermia) and the reprogramming of the immunosuppressive tumor microenvironment (TME) (e.g., M2-to-M1-like macrophages repolarization of tumor-associated macrophages (TAMs)) are particularly appealing to enhance the efficacy of approved immunotherapies (e.g., immune checkpoint inhibitors, ICIs). Due to their modular construction and versatility, iron oxide-based nanomedicines such as superparamagnetic iron oxide nanoparticles (SPIONs) can combine these different approaches in a single agent. SPIONs have already shown their safety and biocompatibility and possess both drug-delivery (e.g., chemotherapy, ICIs) and magnetic capabilities (e.g., magnetic hyperthermia (MHT), magnetic resonance imaging). In this review, we will discuss the multiple applications of SPIONs in cancer immunotherapy, focusing on their theranostic properties to target TAMs and to generate MHT. The first section of this review will briefly describe immune targets for NPs. The following sections will deal with the overall properties of SPIONs (including MHT). The last section is dedicated to the SPION-induced immune response through its effects on TAMs and MHT.

## 1. Introduction

Cancer ranks as a leading cause of death and an important barrier to increasing life expectancy in every country of the world [1]. Cancer is the first or second leading cause of death before the age of 70 years in a vast majority of countries [2], underlining the urgent need to address unmet needs in oncology. According to the type and stage of cancer, various approaches can be employed. While surgery is usually the first line of treatment, other strategies based on chemotherapy and radiotherapy can also be performed. Even if all these strategies can be combined, the desired success rate in cancer treatment has not yet been achieved, especially due to the iatrogenic disorders they induce. As a consequence, many therapies have been developed to specifically and safely target cancers.

Among these targeted strategies, cancer immunotherapies have now revolutionized the field of oncology by prolonging the survival of more and more patients suffering from aggressive and fatal cancers [3]. In immunotherapy, the agents are designed to induce an immune response against cancer cells and can be used in combination, strengthening their central role as a first-line therapy for many cancers in the future. Immunotherapies can be divided into several classes: (i) immune checkpoint inhibitors (ICIs) [4]; (ii) chimeric antigen receptor (CAR) cell therapies (e.g., CAR Natural Killers (CAR NK) [5]; CAR Macrophages (CAR M) [6] and CAR T-Cells [7]; (iii) cytokines-based immunotherapy [8]; (iv) agonistic antibodies against costimulatory receptors [9]; (v) cancer vaccines [10] and (vi) bispecific antibody therapy.

Another very exciting field to specifically and safely target tumors for diagnosis and therapy relies on the use of nanoparticles (NPs), also called nanomedicines. Their size typically ranges between 1 and 100 nm, they can be made from different materials and have various physicochemical properties (e.g., size, shape, surface features, magnetism, etc.). According to their chemical composition, NPs can be classified into organic (e.g., liposomes, polymeric micelles, dendrimers, etc.), inorganic (e.g., super paramagnetic iron oxide NPs (SPIONs), gold nanorods, carbon nanotubes, etc.), or hybrid (e.g., lipid-polymer NPs, organic-inorganic NPs, etc.) NPs [11]. In addition to their intrinsic properties due to the material they are made of, NPs can be modified with a lot of targeting ligands, affecting their biological behavior accordingly.

Even if it is always discussed, it is commonly accepted that NPs target tumors via two main mechanisms. The first one is passive targeting (enhanced permeability and retention (EPR)). There are a few points about the EPR effect that should be made clear. Despite the fact that the EPR effect is frequently described as a process that enables the delivery and retention of drugs at cancerous sites thanks to structural and architectural abnormalities (such as abnormal fenestrations and structural disorganization), the truth is that this increase in permeability and retention is not yet fully understood and may have other explanations. For instance, it is currently understood that this effect, is also influenced by the impairment of lymphatic drainage and permeability-enhancing factors, including nitric oxide, bradykinin, or vascular endothelial growth factors [12]. Moreover, additional phenomena, such as vascular transcytosis-based nutritional pathways (mediated by caveolae, clathrin-coated pits, and macropinocytotic vesicles), may potentially play a role in NP uptake and, subsequently, the EPR effect, especially for NPs with a size between 50 and 100–150 nm [13]. A second transcytosis pathway, known as the vesiculo-vascular organelle (VVO), has also been identified in normal endothelial cells and may potentially contribute significantly to the EPR effect. This system is made up of a vast network of grouped and connected cytoplasmic vesicles and vacuoles. Therefore, more investigation is required to understand exactly the biophysical and metabolic mechanisms that result in the extravasation of NPs into the tumor and, ultimately, the EPR effect [12].

The second mechanism by which NPs target tumors is the active targeting through an ad hoc surface functionalization (e.g., targeting peptide) of the NPs [14]. Through these mechanisms of targeting, NPs are well-known for their capabilities to release encapsulated or conjugated bioactive agents within tumors. NPs make it possible to improve the bioavailability of drugs, to combine therapeutic agents with imaging (i.e., nanotheranostics) techniques, or to boost antitumor effects [15]. Over the last 20 years, around 80 nanomedicine products have been approved by the Food and Drug Administration (FDA) and the European Medicines Agency (EMA) in various indications including cancers [16]. Unfortunately, in spite of considerable technological success, nanomedicines have demonstrated modest effects on survival and in some examples, less than other approved therapies [17].

Since the majority of patients do not benefit from the currently available immunotherapies, and they can experience severe adverse events, immunotherapeutic nanomedicines might enhance efficacy while mitigating certain life-threatening toxicities [18]. Moreover, aiming for pharmacological synergy, it is also possible to design new combinations associating classical immunotherapies and nanomedicines to overcome their respective weaknesses [19]. In this respect, iron oxide nanoparticles (IONPs) such as SPIONs may be an appealing class as they combine many features that allow targeting of the immune system and tumors for theranostic purposes [20]. SPIONs are typically made up of magnetite (Fe_3_O_4_) or maghemite (γ-Fe_2_O_3_) with a core radius ranging from 5 to 15 nm and a hydrodynamic radius (i.e., core with shell and water coat) ranging from 20 to 150 nm [21]. These SPIONs have already been demonstrated to act as advanced platforms for drug delivery and contrast agents in magnetic resonance imaging (MRI) and magnetic hyperthermia (MHT) [22]. Very recently, the theranostic potential of IONPs in cancer immunotherapy has been reported, emphasizing their ability to perform tumor imaging for early assessment of the efficacy of immunotherapy and their capability to alter macrophage polarization [20]. Moreover, more and more studies have demonstrated that SPIONs exhibit the intrinsic capability to stimulate systemic antitumor immune responses through MHT, paving the way for new immunotherapeutic strategies [19].

In this review, we will discuss the multiple applications of SPIONs in cancer immunotherapy, focusing on their intrinsic theranostic properties to target tumor-associated macrophages (TAMs) and to generate MHT in light of their effects on anticancer immunity. The first section of this review will briefly describe immune targets for NPs. The following sections will deal with the overall properties of SPIONs, including the development of MHT. Next, we will see how SPIONs can induce an immune response through the targeting of TME, with a more in-depth focus on TAMs and MHT.

## 2. Immunity, Cancer, and SPION Nanoparticles

### 2.1. Immune Targets in Cancer

#### 2.1.1. Immune Cells and Tumor Microenvironment at a Glance

It is now well-established that immune evasion is a hallmark of cancer [23]. This concept is related to cancer immunoediting, comprising three processes: elimination, equilibrium, and escape [24]. Immune cells are notably present within the TME, a complex network made up of numerous cellular (e.g., vascular, stroma cells) and non-cellular (e.g., extracellular matrix, ECM) components, other than tumor cells. These immune cells (from both innate and adaptive immunity) can either promote or prevent tumor growth. Tumor-promoting immune cells include regulatory T cells (Tregs), myeloid-derived suppressor cells (MDSCs), and tumor-associated macrophages (TAMs). Conversely, tumor-preventing immune cells include CD4+ T helper cells (TH), CD8+ cytotoxic T lymphocytes (CTLs), and natural killer (NK) cells [25]. For a greater insight into the functions of the various immune cells, readers are directed to a comprehensive review conducted by Doshi and Asrani [26]. Since TME is a very immunosuppressive milieu, it seems particularly relevant to pharmacologically activate immune cells (within lymphoid organs or the TME itself) or to target immunosuppressive cells or the combination of these approaches [25]. Interestingly, these strategies can be carried out to potentially target all immune “compartments” (i.e., TME, circulation, and myeloid/lymphoid tissues) since the immunological imbalance in cancer goes beyond the primary tumor [27].

#### 2.1.2. Innate and Adaptive Immunity in Cancer

Both innate and adaptive immunity are crucial components in cancer development and progression and the overall immune response relies on the interplay between them. Innate immunity involves various types of myeloid cells: dendritic cells (DCs), monocytes, macrophages, polymorphonuclear leukocytes (PMNs), mast cells, NKs, and natural killer T (NKT) cells [26]. The innate immune system can directly inhibit tumor progression by engaging tumoricidal activity with NKs (recognition of tumor-derived antigens), granulocytes, and macrophages through antibody-dependent cellular cytotoxicity (ADCC) or antibody-dependent cellular phagocytosis (ADCP) [28]. Conversely, the innate immune system can also contribute to immunosuppression. A major example is represented by protumorigenic M2-TAMs, which express multiple immunosuppressive (e.g., prostaglandin E2, IL10) and tumor-promoting factors leading to suppressed anti-tumor responses [29]. In addition to innate immunity, we find cells from the adaptive immune system, i.e., T-cells and B-cells, whose aim is to eradicate cancer or to inhibit their proliferation through cellular and humoral immunity, respectively. This anticancer response relies notably on the cancer immunity cycle (CIC), a process which can be divided into seven stages starting with cancer antigen release (step 1) and finishing with killing cancer cells (step 7: immunogenic cell death, ICD) through CTLs [30]. Thus, CIC can be self-propagating, leading to an accumulation of immune-stimulatory factors that in principle should amplify and broaden T-cell responses. CIC is also characterized by immune regulatory feedback mechanisms capable of stopping or lowering the immune response. Physiologically, immune tolerance regulating immune responses and preventing tissue damage is mediated by immune checkpoints which are negative regulators of T-cell activation. They refer to immunosuppressive molecules which can be highly expressed in cancer, mediating tumor immune evasion. The main immune checkpoints are cytotoxic T lymphocyte antigen 4 (CTLA-4, expressed on the activated CD8+ and CD4+ T cells), programmed cell death protein 1 (PD-1, expressed in myeloid, B- and activated T cells), and programmed cell death ligand 1 (PD-L1, myeloid and cancer cells). These immune checkpoints have given rise to one of the most important immunotherapies based on their inhibition: the ICIs [31]. LAG3, TIGIT, and TIM3 are other checkpoint signaling molecules, among many others, extensively studied to understand their role in T-cell functions and their potential as new immunotherapies for cancer [32]. It is important to point out that innate and adaptive immunity are tightly connected, notably due to the involvement of antigen-presenting cells (APCs: DCs, macrophages) and complement proteins, resulting in the activation of a T-cell response and immunological memory [26]. This underlines the great interest in using therapeutic strategies capable of targeting both innate and adaptive immunity through, for example, “all-in-one” modalities such as nanomedicines [33], radiotherapy [34], hyperthermia [35], or various synergistic combinations [36,37].

### 2.2. Opportunities for Targeting Immune System with SPION Nanoparticles

#### 2.2.1. Rational for Targeting Immune System with SPION Nanoparticles

As previously mentioned, in spite of breakthrough advances due to immunotherapy for cancer treatment, there is an urgent need to overcome some major limitations. Several reasons can be mentioned to explain some issues related to these treatments [31]. First, there is an intrinsic variability between patients’ immune systems, especially in a context in which they may be immunocompromised by treatments (radiotherapy, chemotherapy), leading to low response rates. Then, as with any cancer therapy, resistance development is inevitable and can be classified as extrinsic (i.e., related to the patient’s gender, TME, gut microbiota) or intrinsic resistance due to the nature of the tumor itself (i.e., “cold” versus “hot” tumors). Finally, safety concerns have been frequently reported through immune-related adverse events (irAEs). Indeed, boosting the innate and/or adaptive immune system has a unique set of inflammatory side effects, which can be life-threatening. In this context, nanomedicines designed to enhance antitumor immunity in a variety of ways might represent an interesting alternative, combining efficacy and safety, alone or in combination with other anticancer strategies [38]. Even if anticancer nanomedicines may enhance tumor targeting, the therapeutic responses cannot be guaranteed, especially when they are used in monotherapy. Indeed, we can observe relapse resulting from the re-establishment of pro-tumorigenic conditions (e.g., progenitor immune cells, re-activation of cancer stem cells) [27]. This underlines the need to develop more holistic approaches, notably based on the immune system, taking into account growth-promoting phenomena that occur inside and outside of tumor tissue. So, according to their design, nanomedicines could take advantage of their numerous properties (e.g., targeting moieties, drug payloads, intrinsic properties such as magnetism) to specifically target the immune system while being able to evade clearance from the bloodstream and reticuloendothelial system (RES). Taken together, these data emphasize the interest in using nanomedicines alone or in combination to target, engage, and modulate immune cells in the TME, circulation, and immune cell-enriched tissues [27].

#### 2.2.2. Nanoparticles to Target Immune TME (iTME)

Very nice and comprehensive reviews related to this topic have been recently published [31,39]. Overall, a lot of different strategies exist in order to target immunity within the TME, especially through its immunosuppressive properties.

The most studied immunosuppressive strategy relies on the inhibition of immune checkpoints, especially in a clinical context with the use of monoclonal antibody-based ICIs targeting CTLA-4, PD1, and PD-L1 [3]. So far, various NPs (organic/inorganic) have been designed with success to deliver ICIs (e.g., siPD-L1, anti-PDL1, anti-PD1, and anti-CTLA-4) in preclinical models [38]. Many other ways to target the immune checkpoints synergistically with ICIs have been performed in combination with various therapeutic modalities such as photothermal therapy (PTT), photodynamic therapy (PDT), radiodynamic therapy, sonodynamic therapy, genetic manipulations, and stimulatory agonists [31]. Another way to remove immunosuppression of the TME is to target indoleamine 2,3-dioxygenase 1 (IDO1), an enzyme producing immunosuppressive metabolites. This enzyme has been shown to be overexpressed in many cancers and many inhibitors have been designed so far. In this context, a prodrug nanoplatorm (approximately 40 nm) has been designed by integrating a PEGylated IDO1 inhibitor (epacadostat) and a photosensitizer (indocyanine green, ICG). Both in vitro and in vivo (B16-F10 cells), the authors demonstrated good efficacy of this strategy, especially in combination with PD-L1 checkpoint blockade [40]. Another way to remove immunosuppression is to reprogram immunosuppressive cells such as M2-like TAMs (*vide infra*) and MDSCs. Phuengkham et al. have targeted both TAMs and MDSCs. They encapsulated resiquimod (TLR7 and 8 agonist) and doxorubicin (to induce ICD) within crosslinked collagen-hyaluronic acid scaffolds. Interestingly, there was subsequent polarization from M2-like to M1-like TAMs associated with the reprogramming of MDSCs into tumor-killing APCs [41].

Another relevant strategy relies on the activation of DCs with nanomedicines. Since DCs are essential to the initiation of the anti-tumor immune responses through the CIC, stimulating their activation has attracted a lot of attention lately. The first strategy to activate DCs is the agonism of the stimulator of interferon genes (STING), a cytosolic receptor mainly localized in the endoplasmic reticulum. When the innate immune system detects DNA from viruses or tumors, cGMAP (an agonist of STING) is produced and activates cells such as DCs, which in turn release type I Interferon (IFN-I). To mimic this mechanism, Wang-Bishop et al. developed a polymeric NP (“polymersome”) encapsulated with cGMAP. These STING-activating NPs were able to induce the expression of IFN-I through DCs stimulation. This was associated with ICD, a remodeling of iTME, and a subsequent inhibition of the tumor growth in neuroblastoma tumor-bearing mice [42]. Another approach to activating DCs relies on cancer vaccines (cellular, protein/peptide, and genetic vaccines), through the use of various NPs. This topic has been recently and extensively reviewed [43].

#### 2.2.3. Nanoparticles to Target Circulating Immune Cells

Before homing on diseased areas, nanomedicines can be recognized and interact with circulating immune cells in the bloodstream. In this case, immune cells loaded with nanomedicines become drug carriers, which significantly extend the circulation time of nanoparticles with broad-spectrum tumor-targeting properties. Interestingly, immune cells can cross many biological barriers and are natural carriers due to their homing characteristics (e.g., inflammatory sites, tumors). To do so, monocytes-macrophages, lymphocytes, and neutrophils might represent the most favorable options. Nevertheless, these cells are quite difficult to extract and are not necessarily optimal for drug delivery (e.g., low drug loading efficiency, changes in cell function after drug loading, and degradation of drugs by cellular enzymes).

According to the loading technique, immune cells are simply divided into two categories: “backpacks” (i.e., onto cells through adsorption, ligand-receptor interaction, and chemical coupling) and “Trojan horses” (i.e., into cells through hypotonic hemolysis, electroporation, membrane encapsulation, and phagocytosis). These different aspects have been extensively reviewed by Zhang et al. [44]. As an example, the Trojan horse strategy has been already used with monocytes as cellular vehicles for the co-transport of oxygen-loaded polymer bubbles/a photosensitizer (chlorin e6) and SPIONs to target hypoxic tumors with photodynamic therapy (PDT). Following activation by an external high-frequency magnetic field (HFMF), the co-entrapped SPIONs induced the thermal ablation of murine prostate (Tramp-C1 tumor-bearing mice) while inducing the release of oxygen available for the PDT effect [45].

#### 2.2.4. Nanoparticles to Target Myeloid and Lymphoid Immune Cell-Enriched Tissues

Nanomedicine is well-known to be uptaken by the RES, and various strategies have been documented to overcome this major drawback [46]. RES is a part of the immune system composed of phagocytic cells found in the spleen, liver, lungs, bone marrow, and lymph nodes. So, it is important to consider that targeting immune cells also implies delivering drugs to these immune-cell-rich organs [27]. Nevertheless, this apparent drawback may be advantageous to target immune cell-enriched tissues for both diagnosis and therapy. Indeed, in various cancers, nanoparticles are administered subcutaneously to target lymph nodes for preoperative imaging and intraoperative detection (radioactivity, fluorescence, magnetism).

As an example, a novel mannose-labeled SPION was recently developed (maghemite iron oxide core) to target lymph node resident macrophages, making it possible to perform lymph node imaging in pigs with a substantial percentage of accumulated iron (83%) [47]. Moreover, it is also possible to target RES with NPs to elicit a personalized anti-cancer response through various lipid NP platforms, allowing the targeted delivery of mRNA or gene editing in a tissue-specific manner [27].

## 3. SPIONs: An Overview

### 3.1. Biophysical Properties of Superparamagnetic Materials

In this section, we introduce the notion of magnetism and present the specific advantages of superparamagnetic materials for MRI and hyperthermia treatment.

All matter exhibits magnetic properties [48,49,50]. However, purely diamagnetic materials made of atoms with filled electron shells exhibiting no magnetic moment must be distinguished from atoms containing unpaired electrons generating magnetism. In other words, the electronic configuration of atoms and the collective behavior of individual atomic magnetic moments in a material allows us to classify materials into different magnetic types, as summarized in Table 1.

#### 3.1.1. Magnetic Behavior of Ferromagnetic and Ferrimagnetic Materials

Ferrimagnetism and ferromagnetism are the magnetism types of interest for medical or industrial applications, thanks to the strong magnetic response they provide. Ferromagnetic and ferrimagnetic materials show a similar temperature dependence of magnetization and the ability, under particular conditions, to exhibit a non-zero net magnetization at zero magnetic fields [48].

The ferromagnetic materials (e.g., Fe, Ni, or Co) are characterized by strong (negative) exchange interactions which are opposed to the thermal agitation effect [51]. As a consequence, the atomic magnetic moments undergo a parallel self-alignment inducing a spontaneous magnetization even in the absence of an external magnetic field. Ferrimagnets are characterized by an anti-alignment of atomic magnetic moments of non-equal magnitudes. Iron oxides such as magnetite (Fe_3_O_4_) and ferrites are examples of ferrimagnetic materials [48,52]. The spontaneous magnetization of these materials remains true below the Curie temperature (Tc) for ferromagnets and below the Néel temperature for ferrimagnets [51,53,54]. Above these critical temperatures, the thermal energy overcomes the exchange interactions and the material is then a paramagnet [48].

The understanding and observation of the microscopic magnetic structure of ferromagnetic materials began in the middle of the 20th century [50,55,56]. Ferromagnetic materials are organized in small regions within which the magnetic moments are aligned in parallel, whereas the net magnetization of all regions is null in the absence of an external magnetic field. These regions, called magnetic domains, are separated by micrometric magnetic walls in which the orientation disrupts by 90° or 180° [51]. In the absence of a magnetic field, every domain can have a specific orientation [57,58]. The domain formation relies on a combination of exchange interactions and other contributions such as magnetostatic energy (the energy inherent to time-independent magnetic fields) allowing minimization of its total magnetic energy [50,51,55].

**Figure 1:**
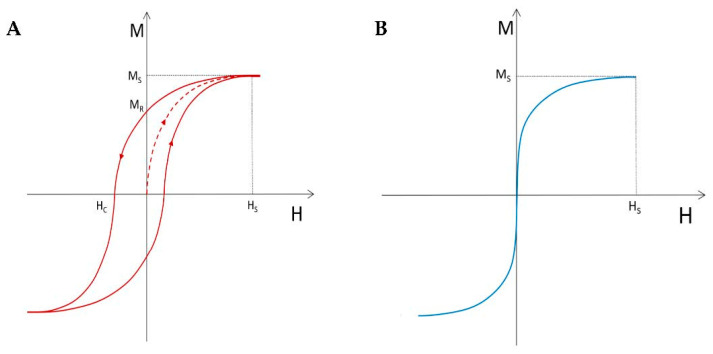
(**A**) Schematic illustration of the typical hysteresis curve of a ferromagnetic material. Starting at field H = 0, M increases towards the saturation magnetization M_S_ (dotted line) and then decreases following a non-reversible path. M_R_ represents the remanent magnetization obtained when H reaches zero. H_C_ represents the coercivity, i.e., the field to apply to nullify the magnetization. The open loop area represents the hysteresis energy losses in the material during the reversal process (heat production). (**B**) Typical magnetization curve of a superparamagnetic material, characterized by the absence of coercivity and hysteresis.

The external magnetic field progressively forces the domains to align in the direction of the field. As a consequence, the domains with a direction close to the applied magnetic field grow to the detriment of others, until all domains finally align in this direction [51]. At this stage, saturation magnetization is achieved [54]. Ferromagnets and ferrimagnets exhibit a non-linear relation between the applied magnetic field intensity H and the resulting magnetization M. M depends on the history of the applied magnetic field [59], or in other words, the magnetization curve of a material does not follow the same path when applying and removing the external magnetic field. Plotting M versus H leads to a hysteresis loop, reproducible in consecutive H cycles [58]. A part of the magnetic moment alignment remains after the magnetic field is removed. This is expressed by the remanence value M_R_ [48,54] located at the intersection of the hysteresis curve with the ordinate axis in Figure 1. To nullify magnetization, a reverse magnetic field must be applied, reported by the coercivity coefficient [52].

#### 3.1.2. From Ferromagnetic and Ferrimagnetic to Superparamagnetic Behavior

As the size of the particles composing a ferromagnet or ferrimagnet decreases, the amount of energy required to create domain walls in this material increases. Below a critical diameter, the coercivity of the material tends to zero due to the anisotropy energy reduction [51]. When the ferromagnetic or ferrimagnetic particle diameter is small enough (of the order of 100 nm or smaller [57,60,61]), and while the thermal energy overcomes the anisotropy energy, the assembly of individual spin magnetic moments behaves as a single super-spin [62] and the particle exhibits a single magnetic domain structure [52,57]. Although the existence of single-domain ferromagnets was predicted in the 1930s [63], important theoretical advances notably by Néel [64], and new measurement methods were required before applications based on this particular magnetism type emerged [65].

In the absence of an external magnetic field, the single-domain magnetization direction is determined by the magnetocrystalline anisotropy of single-domain nanoparticles, which represents the easy (preferred), intermediate, and hard magnetization directions [51]. In the case when the net magnetization of single-domain particles flips randomly very fast under the influence of thermal fluctuations, the magnetization is nulled [52]. When a magnetic field is applied to them, as shown in Figure 1B, the super-spins of individual particles align in the direction of the magnetic field, and the net magnetization increases rapidly and saturates: a behavior shared with paramagnetism. However, unlike paramagnets, materials of this type have a very high magnetic susceptibility due to the ferromagnetic or ferrimagnetic nature of the super-spin. This remarkable behavior is referred to as superparamagnetism [66]. It must be noted that not all single-domain particles are concerned with superparamagnetism [48].

Superparamagnetic materials exhibit remarkable biophysical properties that have been exploited in the medical field since the late 1970s for diagnostic and therapeutic applications [67,68,69,70,71,72]. Firstly, their interaction with the protons of water molecules allows them to be used as a contrast agent in MRI. Secondly, when excited by an alternative magnetic field (AMF) at the appropriate frequency and amplitude, they release thermic energy, which led to the development of MHT, suitable for cancer treatment. More generally, they lose their magnetism when the external magnetic field is removed [73]. In addition to their physical properties, the biocompatibility of iron-based particles explains their increasing use in medicine. Indeed, the human body is able to handle, store and eliminate iron, which is used in several physiological processes such as oxygen transport, DNA synthesis, energy production, and metabolism [66].

The biomedical potential of superparamagnetic materials led to the emergence of a new class of biocompatible superparamagnetic agents referred to as SPIONs, or ultra-small SPIONs when their size is up to 50 nm, monocrystalline iron oxide (MION) between 10 nm and 30 nm [74], or sometimes SPIO (for superparamagnetic iron oxide particles) for particles having a diameter greater than 50 nm [75]. For simplification purposes, the appellation of SPION will be used hereafter to designate all magnetic nanometric particles.

Each SPION consists of a core containing water-insoluble magnetite or maghemite crystals made of thousands of paramagnetic Fe ions [75,76] encapsulated in a biodegradable coating, which strongly influences the magnetic properties of the agent. In addition to preventing the aggregation of SPIONs, which increases the risk of vascular embolism, the coating is used to target specific tissues and thus direct its biodistribution. Particles are suspended in a biocompatible fluid before being administered to a subject. The amount of iron ions contained in a single nanoparticle explains the high contrast capabilities of SPION-based agents [61].

### 3.2. Overview of the Use of SPIONs as MRI Contrast Agents

MRI is a powerful imaging modality for soft tissue imaging as it offers high spatial resolution and tissue discrimination without exposing the subject to ionizing radiations. Hydrogen protons are abundant in our bodies. The proton resonance is obtained by the application of short radio frequency (RF) pulses changing their magnetic moment orientation. After the RF is stopped, relaxation occurs, and magnetic moments realign along their original alignments. The reorientations of spins along the B0 axis, i.e., the spin-lattice relaxation, is characterized by the relaxation time T1, the inverse of which is the relaxation rate R1 = 1/T1 (expressed in s^−1^ or Hz). The disappearance of the magnetization in the transverse plane, named spin-spin relaxation, is characterized by the relaxation time T2 (or relaxation rate R2 = 1/T2). The relaxivity (usually expressed in s^−1^mM^−1^ or L.mmol^−1^.s^−1^) expresses the R1 or R2 proton relaxation rate modulation induced by an MRI contrast agent in a biological tissue as a function of its concentration.

SPIONs are commonly employed as the contrast agent to change the tissue relaxation rates of normal or pathological tissues in order to improve the sensitivity and specificity of MRI. SPIONs administered for this purpose should offer high relaxivity and adequate biodistribution without inducing local or systemic toxicity. The effect of SPIONs on T1 and T2 relaxations depends on both the saturation magnetization of the nanoparticles and their interaction with water protons in tissues. The size, shape, and surface coatings of SPIONs strongly modulate their T1 and T2 effects [61]. The physical phenomena resulting in the modification of the spin-lattice and spin-spin relaxation rates in tissues under the influence of SPION nanoparticles are thoroughly detailed elsewhere in the literature [61,75,76]. Briefly, the accumulation of SPIONs in a tissue induces local perturbations of the principal magnetic field of the MRI system, which increase spin dephasing and finally shorten the transverse relaxation time (T2). This arises from the magnetic coupling between protons spin in tissues and the spins of SPIONs. Therefore, the presence of SPIONs causes a negative MRI contrast in tissues.

T2-shortening agents have two major drawbacks: (1) the increased magnetic susceptibility artifacts and (2) the difficult interpretation of low-signal areas which may be confused with bone or vascular structures [77]. This encouraged the development of particles providing contrast in both T1-weighted (T1w) and T2-weighted (T2w) imaging. For instance, gadolinium-labeled magnetite nanoparticles dedicated to positive contrast MR angiography were successfully used in vitro and in vivo by Kellar et al. [78]. More recently, a new class of cubic SPIONs, suitable for use as a dual-mode contrast agent, was presented by Alipour et al. [79]. Although these agents do not yet represent the majority in the literature, their development has been accelerated in recent years, expanding possible diagnostic applications with SPIONs. The typical R2 relaxivity of SPIONs ranges from 100 s^−1^mM^−1^ to a few hundred s^−1^mM^−1^ depending on the characteristics of the particle (composition, coating) and the B0 MRI field [61]. One of the challenges of current studies is to increase the R1 relaxivity) (usually much lower than R2 relaxivity).

The oral administration of SPIONs as a gastrointestinal MRI contrast agent has been considered by several research teams [76]. For instance, Hahn et al. described the improvement of the gastrointestinal tract delineation of MR images provided by a 200 nm SPIO suspended in a low-viscosity food-grade fluid [80]. Their preparation was globally well tolerated by animals and patients. Apart from a few special cases, in the vast majority of studies on the subject since the late 1980s [75], SPIONs are administered intravenously [75]. Unlike low molecular weight water-soluble agents such as gadolinium chelates, SPIONs are usually not transferred to the extracellular-extravascular compartment in healthy subjects and are rapidly eliminated by the RES [67,69,70]. As a consequence, their biodistribution is characterized by a short biological half-life and a significant accumulation in the RES (typically: liver, spleen, bone marrow). As a first approach, SPIONs can thereby be used to enhance malignant lesions within organs of RES [80]. For instance, Weissleder et al. used SPIONs to detect focal splenic tumors with MRI, leading to an important step forward in this domain since the other existing imaging techniques do not provide contrast between such lesions and healthy tissues [67].

On the other hand, the phagocytosis of SPIONs allows the visualization of tissues infiltrated by macrophages during inflammatory processes, which would not be possible with gadolinium-based contrast agents, not internalized by immune cells [81]. Macrophages are the key component of acute inflammation [35]. When an infectious agent is detected, an immune response is set up resulting in vasodilation, higher vascular permeability, and infiltration of free fluid and immune cells (neutrophils and macrophages) in tissues [81,82]. These phenomena are followed by the formation of a fibrotic scar. MRI procedures taking advantage of the phagocytosis of SPIONs for the detection of inflammatory areas and infectious foci, and more generally for the assessment of immune-mediated disorders, were described in the mid-2000s [82,83]. Stoll et al. described the interest in SPIONs in the assessment of central nervous system inflammations [84]. Sillerud et al. detected amyloid-β plaques in a transgenic mouse model of Alzheimer’s disease [85]. Ruehm et al. described, in a preclinical assay, the interest in SPIONs as a marker of atherosclerosis (chronic inflammatory response to a vascular wall injury) [86].

Over the last few decades, significant progress has been made in the design of SPIONs, such as the reduction in the average size of these nanoparticles, the improvement of their physico-chemical characteristics, the incorporation of innovative coatings, and especially their surface functionalization. For instance, by decreasing the diameter of their SPIONs to the size range of plasma proteins (i.e., around 10 nm), Weissleder et al. increased their biological half-life and facilitated their transcapillary passage to the interstitium. As a result of these improvements, SPIONs were progressively promoted to the rank of multimodal theranostic nanoprobes with, among others, applications in MHT treatment and immunotherapy. It has been established that MRI examinations performed after the administration of SPIONs offer higher sensitivity and specificity than non-injected MR acquisitions in the diagnosis of lymph node metastasis [87].

SPIONs can also help to distinguish infectious masses from cancerous tumors [81]. Preclinical [88] and clinical studies attested to the benefits of such examinations in axillary node metastases detection in breast cancer patients [87]. Other authors proved the interest in SPIONs for cardiovascular system explorations. Majundar et al. showed the blood-to-background nuclear magnetic resonance (NMR) signal ratio improvement provided by a 72 nm SPION used in rat brain perfusion imaging [72]. Antonelli et al. reported the use of SPIONs to image atrioventricular fistulas, chronic venous occlusions, and lower extremity arteries [74]. SPIONs internalized by macrophages were also reported as a possible contrast agent of the vascular phase, providing cardiovascular applications such as perfusion and viability imaging [75,78,89]. Moreover, for assessing the inflammatory microenvironment of primary/metastatic tumors and for monitoring the therapeutic response of cancer patients receiving radiotherapy and immunotherapy, non-invasive imaging of TAMs with SPION may offer considerable potential [90].

Limitations to the SPION-enhanced MRI examinations have been mentioned in the literature [81,91]. These imaging procedures are long compared with other imaging modalities, potentially causing a greater motion sensitivity. The concentration of MR probes must reach 0.01 mM to 10 mM for efficient detection [92]. By comparison, in single photon computed emission computed tomography (SPECT) and positron emission tomography (PET) imaging, the tracer can be detected at the picomolar scale [93]. Increased iron levels in the body can lead to tissue damage through oxidative injury. This must be taken into account for repeated examinations or longitudinal studies [81], or if the iron clearance rate of the subject is altered. Particles remaining after a SPION-enhanced acquisition can also cause major susceptibility artifacts and interfere with other MR acquisitions even several months after the injection for liver MRI [81].

### 3.3. Basic Aspects of Magnetic Hyperthermia (MHT)

#### 3.3.1. Biological Aspects of Hyperthermia

Tumoral tissues potentially contain necrotic, hypoxic, and low pH areas rendering them resistant to chemotherapy and radiotherapy. Moreover, cells in late phase S (Synthesis i.e., DNA replication) are usually more radioresistant than cells in the M phase (Mitosis) but sensitive to heat [94]. In this context, hyperthermia in conjunction with conventional therapies such as chemotherapy drugs or radiotherapy brings a synergistic therapeutic effect [53] and potentially improves tumoral regression [94]. The therapeutic effect of hyperthermia relies on the fact that cancer cells are more sensitive to heat because of their increased metabolic rate [53]. The exposition of cancer cells to a 40–46 °C temperature induces a thermal shock modifying cellular processes, altering the structure and function of proteins and ultimately promoting apoptosis of exposed cells. In addition, heat stress restores blood flow, permeability, pH, and oxygenation of the tumor microenvironment [53,73] and inhibits the repair of ionizing radiation-induced DNA damage [48]. Moreover, MHT may induce effective and genuine immunogenic tumor cell death as recently demonstrated [95]. MHT is the main hyperthermal strategy currently being developed for therapeutic applications. An optimal hyperthermia treatment allows a high heating efficiency, in a short time, and with a minimum concentration to avoid systemic side effects [54].

#### 3.3.2. Heat Production Mechanisms in MHT

MHT relies on the conversion of magnetic energy into thermal energy by the action of an alternative magnetic field with a frequency usually ranging from 100 to 300 kHz and a moderate amplitude [53,54,66]. Four independent mechanisms contribute to heat production: eddy current loss, hysteresis loss, Néel relaxation loss, and Brown relaxation loss [48,53,57]. The relative contributions of these four effects are determined by particle size, magnetic anisotropy, and fluid viscosity. The specific absorption rate (SAR) expressed in W/kg, quantifies the thermal power dissipation. SAR increases in proportion to the thermal energy released in the material [57]. The physical basis of MHT is well described elsewhere [48,54,57,96].

### 3.4. Design of SPIONs

#### 3.4.1. General Design of Cancer Nanomedicines

Concerning the general design of a given nanomedicine, the function, characteristics, materials, and method of synthesis are the four factors that must be taken into account. Once clearly established, the defined function is then used to specify the essential characteristics of the nanomedicine. Then, the materials used to build the object and its manufacturing method have a significant impact on its ability to exhibit the desired properties [14]. Thus, the physicochemical properties obtained from a rational design will have a major influence on the biodistribution and pharmacokinetics of a nanomedicine. The major physicochemical properties of a given nanomedicine are the size, the shape, the surface charge, and chemistry. The size of nanomedicines is a major feature dictating their overall biodistribution and cell internalization. As previously mentioned, the suitable diameter for nanomedicines targeting cancers ranges from 10 to 100 nm, making it possible to passively target tumors through the EPR effect.

The surface charge, expressed through zeta potential (i.e., the surface charge of an NP in colloidal suspension), is also a major parameter related to nanomedicine biodistribution due to electrostatic interactions between the NPs and various biological molecules. Unlike negatively charged NPs, positively charged NPs are more prone to be uptaken by cells that may induce side effects (non-targeted cells), decrease a drug delivery process to targeted cells, and shorten the circulation time. Zeta potential may also affect the loading capacity of SPIONs according to the charge of the payload drug. The surface chemistry of nanomedicines aims also to significantly improve their targeting properties. This goal can be achieved through various functionalizations with targeting ligands enhancing the affinity of nanomedicines to significantly increase the uptake within the targeted tissues. These functionalizations can also rely on surface modification with various polymers (e.g., polyethylene glycol, PEG, *vide infra*) to protect the nanomedicines from rapid clearance, subsequently making them stealthy. The clearance phenomenon may also be increased through renal elimination. This can be observed with small NPs (approximately 6 nm), positively charged NPs (through the negative glomerular basement membrane), and rod-shaped NPs [14].

#### 3.4.2. Design of SPIONs Suitable for Hyperthermia and Immune System Targeting

SPIONs can be produced through physical, biological, or chemical routes. Nevertheless, chemical syntheses remain the main way to obtain SPIONs. The co-precipitation method has been the starting point for other approaches such as thermal decomposition methods, hydrothermal methods, solvothermal methods, sol-gel methods, micelle methods, and many other methods [97,98,99].

The method of synthesis drives the size, shape, colloidal stability, and magnetic properties of the SPIONs. For biomedical applications, SPIONs need to be modified to enhance their stability. This goal can be achieved through the grafting of various polymers such as PEG, polyethyleneimine (PEI), polyacrylic acid (PAA), polyvinylpyrrolidone (PVP), dextran, chitosan, and many others [97].

For instance, polymers such as PEG are now well-known to increase the biocompatibility, colloidal dispersion, and stability of SPIONs while conferring them relative stealthiness towards the RES. [100]. Silanes are also common coating polymers of SPIONs, used for example to modify the surface (aminosilane type shell) of NanoTherm^®^ (size about 15 nm). This is the only SPION approved to treat glioblastoma with MHT induced with AMF [97]. Nevertheless, it is important to underline that NanoTherm^®^ has to be injected directly into the tumor.

Many interesting preclinical/clinical studies on magnetic hyperthermia (MHT) with SPIONs have been carried out, but very few have led to advanced clinical phases [101]. In spite of its well-known efficacy on cancer cells, the main drawback of hyperthermia is its lack of selectivity between healthy and tumor tissues. To overcome this issue, SPIONs might be one of the most promising solutions if tumor targeting techniques are used (i.e., intra-tumor implantation, pharmacological targeting, and/or magnetic field application). In this context, several clinical trials have been performed with SPIONs, especially in the field of prostate cancer and glioblastoma [102]. Even if most studies have proven good efficacy, methodology/instrumentation issues impair the broader use of magnetic hyperthermia. Nevertheless, the case of Nanotherm^®^ is particularly interesting and gives rise to much hope in this field. Indeed, it is the only approved nanoparticle for MHT of glioblastoma (CE marking, approval in 2010 as a class III medical device) and has recently (2021) achieved a new accomplishment in prostate cancer, being allowed by the FDA to move towards a pivotal phase 2b clinical trial.

So, we could consider that the year 2021 is likely to be a turning point for MHT with the arrival in early clinical phases of new iron nanoparticles from the NoCanTher project (RCL-01, a 149 nm iron oxide nanoparticles coated with dextran) to treat locally advanced pancreatic ductal adenocarcinoma [103]. Based on their designs, we can consider that these products developed and used in clinical settings belong to the first generation of SPIONs. Indeed, these SPIONs are based on a magnetic core decorated with an organic coating without any targeting moieties. This justifies their implantation in situ with surgical procedures to achieve MHT with ad hoc devices. Moreover, the cancers addressed by these new therapies are well-known to be particularly challenging from a pharmacokinetics point of view (blood-brain barrier, blood-prostate barrier), justifying once again, the intratumor injection. We could consider that the intratumoral delivery of SPIONs for MHT is the counterpart of what is classically done in the framework of brachytherapy to treat many cancers (gynecological, prostate, and skin).

Based on these recent data, we assume that MHT with SPIONs is still in its infancy, paving the way to future smarter approaches. Thus, the modularity and theranostic capabilities of SPIONs should make it possible to design and develop a new generation of tumor-selective drugs until clinical phases. Ideally, this new generation should be suitable for the intravenous route with an optimal tumor uptake guided with MRI, allowing us to perform a safe and efficient MHT procedure.

So, for reasons of therapeutic refinement, SPIONs may also acquire active targeting capabilities through, for example, surface modification with antibodies, targeting peptides, or any other molecules with biological targeting capability [98]. However, it should be remembered that the changes in the surface of SPIONs may modulate the thickness of the overall surface coating, affecting the performances of T2 relaxation (MRI) and MHT [104,105]. Overall, when designing SPIONs for MHT, a balance must be achieved between the size of the magnetic core to maximize heat release (>10 nm) and colloidal stability in biological media required for intravenous injection (ideally <50 nm) [106]. Shape is also a major parameter to take into account when designing SPIONS for MHT purposes. For example, cubic-shaped SPIONs (from 17 to 61 nm) have been found to be more efficient in vitro to induce MHT when compared to spherical ones [107]. This effect was verified in vivo in subcutaneous A431 tumor-bearing mice, showing that cubic-shaped SPIONs coated with a polymer shell were able to induce effective MHT and heat-mediated chemotherapy [108].

Immune cells involved in the immunotherapy mechanism can be targeted with SPIONs. Thus, with a more or less sophisticated design based on the strategies previously evoked, SPIONs can be used for cancer vaccines, the guidance of magnetized cytotoxic cells to tumor sites, drug delivery of immune checkpoint inhibitors, the polarization of macrophages, and to trigger magnetic hyperthermia [109]. Of course, the modular construction of SPIONs and their magnetic properties allow us to consider combinatorial immunotherapies in the same nanomedicine [110]. Below, we will emphasize these approaches with recent studies given as examples of SPIONs designed for immunotherapeutic uses. For cancer vaccines, strategies based on ovalbumin bound to SPIONs (size around 200 nm, zeta potential around −22 mV) have been successfully evaluated as a vaccine delivery platform and immune potentiator, showing the activation of immune cells and cytokine production [111]. SPIONs can also be used as platforms to magnetically guide immune cells such as T cells to a region of interest. To do so, Ortega et al. designed several SPIONs coated with dimercaptosuccinic acid (DMSA), 3-aminopropyl-triethoxysilane (APS), or dextran (6 kDa). The size of these SPIONs ranged from 82 to 120 nm (zeta potential from −34 to +38 mV) and made it possible to activate in vivo the migration of T cells, loaded with SPIONs, through the application of an external magnetic field [112].

Another major way to target immunity with SPIONs is to target immune checkpoints since they are becoming a standard regimen in oncology. Very recently, Kiani et al. designed sophisticated SPIONs (90 nm, zeta potential of 28.6 mV), covered by chitosan, functionalized with TAT peptide (cell-penetrating peptide) and loaded with siRNA to silence two of the most important T-cell immune checkpoints (PD-1 and A2aR) [113]. These SPIONs significantly inhibited tumor growth (in CT26 and 4T1 mouse tumors) associated with an important anti-tumor immune response and survival time. SPIONs can also be designed to induce the repolarization of M2 to M1 (*vide infra*). In this way, Zhang et al. perform a study with differently charged SPIONs in order to see potential preferential differences in polarizing macrophages [114]. They synthesized three differently charged SPIONS (zeta potentials of +44.72 mV, −0.282 mV, and −27.31 mV for sizes about 19.4 nm, 15.9 nm, and 21.3 nm, respectively). Interestingly, they demonstrated that positively charged SPIONs had the highest cellular uptake and higher macrophage polarization effect (i.e., M2-like macrophages toward M1-like macrophages).

The shape of SPIONs is also an important parameter affecting the immunological response. Among the various existing shapes (e.g., spheres, rods, cubes, etc.) that have been designed so far, octapod- and plate-shaped SPIONs showed a higher immunomodulatory potential. The shape also influences the targeting and uptake within immune cells. For example, the internalization of spherical SPIONs is increased when compared to non-spherical ones. Conversely, at similar size and charge, spherical SPIONs are less efficient at diffusing across the vascular wall when compared to rod- or bar-shaped SPIONs [110].

In the context of immunotherapy, SPIONs are particularly suitable platforms for theranostic combinations. In this way, Wang et al. designed spherical SPIONs suitable for MRI, targeting M2-like macrophages and MHT in breast tumor-bearing mice [115]. They obtained a multifunctional SPION (hydrodynamic diameter of 20 nm), with efficient targeting capability, high relaxivity (149 s^−1^mM^−1^), and satisfactory magnetic hyperthermia performance in vitro. In vivo MRI showed that M2-targeting SPIONs had a good biodistribution within tumors, also indicating the optimal timing for MHT. The MHT procedure induced both a decrease in the population of M2-like TAMs and tumoral volume associated with iTME remodeling (notably through a significant increase in CTLs). To go further, we invite the reader to consult a recent review related to the enhancement of CD8+ T-Cell-Mediated tumor immunotherapy via MHT used alone or in combination [116].

**Figure 2:**
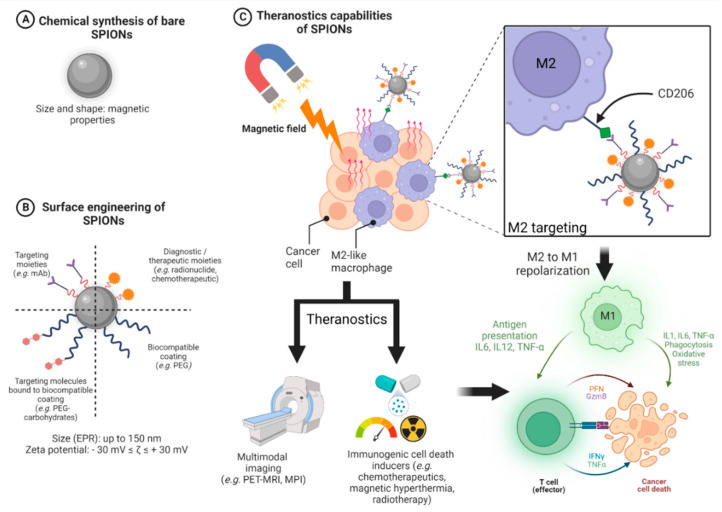
Design and theranostic applications of SPIONs suitable for MHT and macrophage targeting in cancers. Abbreviations. PEG: Poly-Ethylene Glycol; mAbs: monoclonal antibodies; EPR: Enhanced Permeability and Retention; MRI: Magnetic Resonance Imaging; MPI: Magnetic Particle Imaging; PET: Positron Emission Tomography; IL: Interleukin; TNF-α: Tumor Necrosis Factor-α; AMF: Alternating Magnetic Field; PFN: Perforins; GzmB: Granzyme B; IFNγ: Interferonγ. Created with BioRender.com.

Due to the intrinsic versatility of nanomedicine, the various data in the literature show that there is no real consensus on the design of SPIONs. This suggests that the design of a given nanoparticle must be thought of in terms of its future application allowing us to imagine the most suitable specifications resulting from an optimal design. Figure 2 summarizes the design process of theranostic SPIONs emphasizing MHT and targeting M2-like tumor-associated macrophages. The first of these steps (Figure 2A) is the synthesis of the magnetic core (bare SPIONs), which influences its magnetic properties. Unless there is a magnetic field, magnetization equals 0. The core radius usually ranges from 5 to 15 nm. Many synthesis methods are available and drive the convenience of manufacturing, the control of shape, size, composition, and the polydispersity index (i.e., estimation of the average uniformity of a nanoparticle solution) of SPIONs. The second step is the surface engineering of SPIONs (Figure 2B). SPIONs can be coated with various organic moieties for biocompatibility (e.g., PEG, chitosan), targeting (e.g., mAbs, peptides), and theranostic (e.g., radionuclides, chemotherapeutics) purposes. Targeting molecules (e.g., carbohydrates such as mannose to target M2-like CD206 receptors) can also be bound to the biocompatible moieties. The surface engineering will influence the hydrodynamic size (i.e., core with shell and water coat—typically between 20 and 150 nm), zeta potential (i.e., the electric charge on the surface of a given nanoparticle, crucial for colloidal stability, typical absolute value: |30| mV), cellular uptake, toxicity, and hydrophilicity. Size also influences the EPR effect (i.e., passive targeting of tumors, up to 100–150 nm). Finally, the theranostic capabilities of SPIONs are assessed (Figure 2C). Due to their intrinsic superparamagnetic properties, the application of a magnetic field makes it possible to perform MRI, MPI, and MHT (with AMF) and concentrate SPIONs within tumors. Interestingly, decorated SPIONs can target tumors and their microenvironment (e.g., M2-like macrophages through their CD206 receptor) to either exert their diagnostic (MRI, multimodal imaging such as PET-MRI, MPI) and/or their therapeutic (MHT, drug delivery) properties according to the design. In the context of immunotherapy, SPIONs might be particularly appealing through the combination in the same agent of immunogenic cell death inducers such as MHT and/or other thermal/phototherapies (e.g., photothermal therapy, photodynamic therapy), chemotherapy (e.g., doxorubicin), and radiotherapy in addition to macrophage repolarization from M2 to M1 phenotype. This combination makes it possible to boost both innate and adaptative immunity against tumors through the production of various tumoricidal mediators (cytokines such as IL1, TNF-α, and reactive oxygen species). Overall, in addition to these outstanding theranostic properties, SPIONs possess other many advantages such as long-term chemical stability, biocompatibility, and safety. Nevertheless, especially for MHT, the targeting strategies need to be improved to achieve a high concentration of SPIONs within targeted tissues to significantly reduce non-specific heating and increase efficacy. Moreover, in the context of clinical perspectives, all metallic material within 40 cm of the treatment area must be removed prior to alternating magnetic field exposure [117,118].

## 4. Targeting the Immune System with SPIONs

### 4.1. Magnetic Hyperthermia Based on SPIONs as an Immune Trigger against Tumors

Cancer cells are more sensitive to hyperthermia (elevation of temperature to 40–45 °C) than normal cells [119,120,121]. This may be because cancer cells have a more accelerated metabolism [122] or because there is poor vascular distribution in cancerous tissue, leading to an accumulation of fever and heat stress [123].

In this sense, several methods of increasing the temperature in order to eradicate tumors have been investigated, such as those based on radiofrequency, microwaves, or ultrasound [124]. It is in this context that SPIONs can be used to generate heat via the use of electromagnetic energy, the so-called MHT [125]. Indeed, as previously seen and thanks to their magnetic properties, when subjected to an AMF, SPIONs are able to produce heat [118]. Furthermore, since SPIONs can be functionalized on their surface with molecules that target cancer cells, it would then be possible to induce localized hyperthermia. This last point is particularly important since a key disadvantage of classical methods of hyperthermia induction is the lack of selectivity [118].

Starting from this premise, only a few clinical trials have been conducted since 2006 to investigate the impact of thermotherapy based on SPIONs on different cancers, mostly glioblastoma and prostate cancer. SPION-based thermotherapy has also been investigated to treat other carcinomas (ovarian, cervical, and rectal) and sarcomas (chondro-, rhabomyo-, and parapharyngeal sarcoma) [126,127,128,129]. In general, these studies have shown that it was possible to have an increase in intratumoral temperature thanks to the combination of SPIONs and AMF. For instance, in prostate cancer, maximum temperatures up to 55 °C were reached [127]. Moreover, in glioblastoma, patients’ overall survival was improved following MHT treatment [129]. In addition, both of these studies highlighted the fact that only moderate side effects were observed, with no serious complications [128,129].

Recently, a phase 0 clinical trial (NCT02033447) investigating SPIONs-induced MHT with AMF has been completed but, as far as we know, no results have been published so far. Interestingly, another recent phase I (NCT04316091) clinical trial will study MHT in osteosarcoma with SPIONs triggered by spinning magnetic fields (SMF, a new type of magnetic field) in association with neoadjuvant chemotherapy [118]. Despite the fact that the feasibility of SPIONs-induced hyperthermia has been demonstrated at both preclinical and clinical levels, the low number of clinical trials can be partly explained by the fact that this thermotherapy is at the interface of several disciplines (physics, chemistry, biology, medicine, pharmacology) with potential issues to in designing ad hoc SPIONs. Therefore, a better understanding of the mechanism of this therapy in preclinical models, including its action on the immune system, is needed. Indeed, beyond the fact that hyperthermia can directly cause cancer cell death by necrotizing tissues [125], this therapy can also indirectly cause cancer cell death by activating antitumor immunity through ICD [124]. In this sense, Persano et al., in the context of glioblastoma, investigated the impact of magnetic hyperthermia on U87 cells in vitro following an iron oxide nanotube treatment. Interestingly, after thermotherapy, U87 cells displayed a different immunological profile (with an increase in stress-associated signals), making them more likely to be phagocyted by macrophages or killed by NK cells [130].

Other recent studies have demonstrated the impact of SPION-based MHT on the immune system. Carter et al. [125] demonstrated in a subcutaneous syngeneic (GL261 cells, glioblastoma) mouse model (C57BL/6), that magnetic hyperthermia treatment following intratumoral injection of Perimag-COOH SPIONs (dextran-coated, negatively charged and with a hydrodynamic diameter about 130 nm), induced an increase in the proportion of CD8+ T cells within tumors, which is a well-known good prognostic factor [131]. Carter et al. also demonstrated in this mouse model that magnetic hyperthermia treatment was able to reduce tumor growth when compared to control groups [125]. Covarrubias et al. showed in another syngeneic (4T1) mouse model (BALB/c), that IONPs-induced hyperthermia decreased immune cell subpopulations, including those from the innate system (such as neutrophils, dendritic cells, and macrophages) and adaptive system (i.e., CD4+ and CD8+ T cells). Interestingly, subsequent treatment with immune checkpoint inhibitors favored tumor repopulation with the infiltration of innate and adaptive immune cells within tumors [132]. More research is needed to fully assess the effects of SPION-based MHT on the tumor microenvironment. Finally, SPIONs may be useful in treating tumors, in addition to their capacity to cause hyperthermia, by reversing the immunosuppressive tumor microenvironment, which includes, among other things, their influence on macrophage polarization.

### 4.2. SPIONs and Immunomodulation of the Monocyte-Macrophage Axis

#### 4.2.1. Solid Tumors and TME

Cancers could be divided into two main types, solid and liquid tumors. Both of them are characterized by uncontrolled cell growth. Whereas liquid tumors, also known as blood cancers, can affect blood cells and their precursors [133], solid tumors can occur in many parts of the body and they can be separated into two major groups according to where they originate: carcinoma (epithelial tissue) and sarcoma (connective tissue) [134]. However, in compliance with Global Cancer Statistics 2020, solid tumors alone account for approximately 90% of adult human cancers [1]. Solid tumors are not only composed of cancer cells. Immune cells, such as B and T lymphocytes or macrophages, as well as non-immune cells, including endothelial and stroma cells, are part of a highly complex ecosystem, which directly interacts with cancer cells, called the TME [135]. The TME is also composed of several non-cellular effectors, such as cytokines, chemokines, and the extracellular matrix (ECM) [136]. Moreover, two key hallmarks of the TME include hypoxia, resulting from anarchic neo-angiogenesis and promoting tumor aggressiveness, and immunosuppression, whereby cancer cells manage to escape from immune cells [137,138].

Immunosuppressive effects observed in the TME are sustained by a group of cells, called immunosuppressive cells, such as regulatory T cells, regulatory B cells, MDSCs, and TAMs [139]. TAMs have an important role in cancer progression as they can account for up to 50% of some solid tumors [140]. The vast majority of TAMs exhibit an immunosuppressive and pro-tumoral M2-like phenotype [141]. However, TAMs can also display an M1-like phenotype that could be correlated with tumor regression [142].

#### 4.2.2. Macrophage Polarization

Two major macrophage phenotypes have been described, the classically activated M1 phenotype, characterized by pro-inflammatory properties, and alternatively the activated M2 phenotype, characterized by an anti-inflammatory and a tolerogenic activity [142]. One of the macrophages’ fundamental features, besides the fact that they display an important phagocytic activity, is their plasticity. They are the most plastic cells of the entire hematopoietic system [143]. In specific terms, macrophages are able to modify their phenotype according to signals perceived in their environment (cytokines, microbial particles, apoptotic bodies, activated lymphocytes) [144]. One of the current challenges in cancer treatment is to find a way to switch TAMs from an M2-like pro-tumoral into an M1-like anti-tumoral phenotype [145].

Among others, the main *stimuli* of M1 polarization are those triggering a pro-inflammatory response such as bacterial wall components (Lipopolysaccharide, LPS, and Lipoteichoic acid, LTA), viruses or cytokines (interferon gamma, IFN-γ, and granulocyte-macrophage colony-stimulating factor, GM-CSF). By contrast, the main *stimuli* promoting M2 polarization include interleukins IL-4, IL-13, IL-10, and the cytokine M-CSF (macrophage colony-stimulating factor), which activate a tolerogenic or even anti-inflammatory phenotype [146].

**Figure 3:**
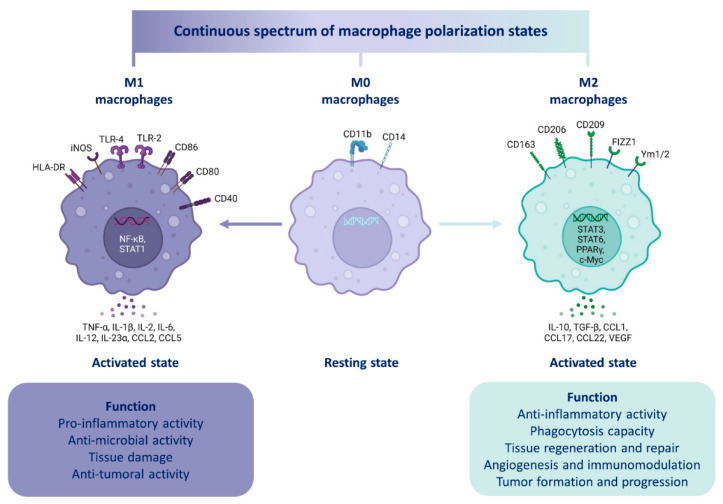
Spectrum of macrophage polarization. Depending on the signals perceived in its milieu (microbial products, damaged cells, cytokines), a naive M0 macrophage can be activated and polarize towards a plethora of different phenotypes. The two extremes of this continuous polarization spectrum are, on the one hand, M1 macrophages, known to have pro-inflammatory activity, and on the other hand, M2 macrophages, known to have anti-inflammatory function. These two extremes were obtained in in vitro models where their polarization markers (such as membrane receptors, transcription factors, cytokines) were identified. However, in vivo, macrophages present in an organism, or in a tumor, will tend towards an M1 or M2 phenotype. Due to the great complexity of the in vivo milieu, these macrophages will never reach the level of polarization that macrophages obtained in vitro. These macrophages in vivo will thus be called M1-like or M2-like [149,150,151,152]. Created with BioRender.com.

This concept of M1 or M2 phenotype (derived from naïve macrophages or M0) is based on in vitro models (Figure 3) where many polarization markers (Table 2) have been identified [147]. Nevertheless, in vivo, given the complexity of the cellular and cytokine environment (specifically in the TME), M1-like or M2-like macrophage terms are preferentially used [148]. M1 and M2 polarization represent two extremes of the macrophage polarization spectrum [142] between which there are various degrees of polarization towards which macrophages are able to converge according to environmental signals and their concentration [149].

Moreover, once a macrophage is polarized, this polarization is not definitive. Thus, depending on environmental signals variation, such as a treatment, an M1-like macrophage may switch to an M2-like phenotype or *vice versa*. This phenomenon, based on macrophage plasticity, is known as repolarization [153]. Therefore, treatments promoting the repolarization of TAMs, such as SPIONs, might be a potential therapeutic lead to inhibit cancer development or even contribute to cancer regression in solid tumors.

**Table 2:** Markers of macrophage polarization.

Molecule Family	Polarization Marker	M0, M1 or M2 Marker	Species	References
Enzyme	Arg1	M2	Murine	[154]
	iNOS	M2	Murine	[154]
Membrane receptors	CD11b	M0	Human/Murine	[155]
	CD14	M0	Human	[155]
	CD40	M1	Human/Murine	[156,157]
	CD80	M1	Human/Murine	[158]
	CD86	M1	Human/Murine	[158]
	CD163	M2	Human/Murine	[155]
	CD206	M2	Human/Murine	[155]
	F4/80	M0	Murine	[155]
Cytokines	IL-1β	M1	Human/Murine	[26,159]
	IL-2	M1	Human/Murine	[26]
	IL-6	M1	Human/Murine	[154]
	IL-10	M2	Human/Murine	[158]
	IL-12	M1	Human/Murine	[26]
	IL-23α	M1	Human/Murine	[26]
	CCL2	M1	Human/Murine	[160]
	TNF-α	M1	Human/Murine	[154]
	TGF-β	M2	Human/Murine	[158]
	VEGF	M2	Human/Murine	[161]

Abbreviations: Arg1: Arginase 1; CCL2: Chemokine (C-C motif) Ligand 2; CD: Cluster of Differentiation; iNOS: inducible Nitric Oxyde Synthase; IL: Interleukins; TGF-β: Transforming Growth Factor beta; Tumor Necrosis Factor alpha; VEGF: Vascular Endothelial Growth Factor.

#### 4.2.3. Macrophage Origin

There are three main cell groups present in peripheral blood, in other words, the blood circulating throughout the body. Erythrocytes and thrombocytes, which are anucleated cells, and leukocytes (or white blood cells), which are nucleated cells that have a role in immunity. Among leukocytes, two subdivisions exist. The first includes granulocytes (including neutrophils, eosinophils, and basophils), which have the particularity of having a multilobed (polynuclear) nucleus, while the second subdivision includes peripheral blood mononuclear cells (PBMCs).

PBMCs include lymphocytes (T, B, and NK), dendritic cells, and monocytes [162]. These circulating monocytes arise from bone marrow, then migrate into tissues through blood and thanks to local signals (essentially cytokines) differentiate into macrophages [163]. These cells, known as tissue-resident macrophages, have a very long lifespan ranging from a few months to years [164]. Tissue-resident macrophages remain in tissues and contribute to their proper functioning (tissue surveillance and clearing) [165].

Furthermore, the differentiation of monocytes into macrophages takes place in two successive steps. First, in a process called maturation, monocytes transform into naïve macrophages (also called M0). Then, in a second step, these cells could be activated and polarized towards a phenotype (M1 or M2) depending on environmental signals [166]. In some organs, such as the gut, the origin and renewal of tissue-resident macrophages rely exclusively upon circulating monocytes [167]. However, the origin and renewal of resident macrophages from other tissues, such as the brain, liver, or lung, is through embryonic precursors produced either by the yolk sac or by the fetal liver. These precursors act as stem cells by ensuring the renewal of these macrophage populations throughout life [168,169].

In tumors, despite there being widespread recognition that TAMs derive predominantly from circulating monocytes, some studies based on murine models of brain, lung, and pancreatic cancers showed that a significant part of TAMs also derived from tissue-resident macrophages [147].

#### 4.2.4. Impact of SPIONs on Monocytes and Macrophages

**Figure 4:**
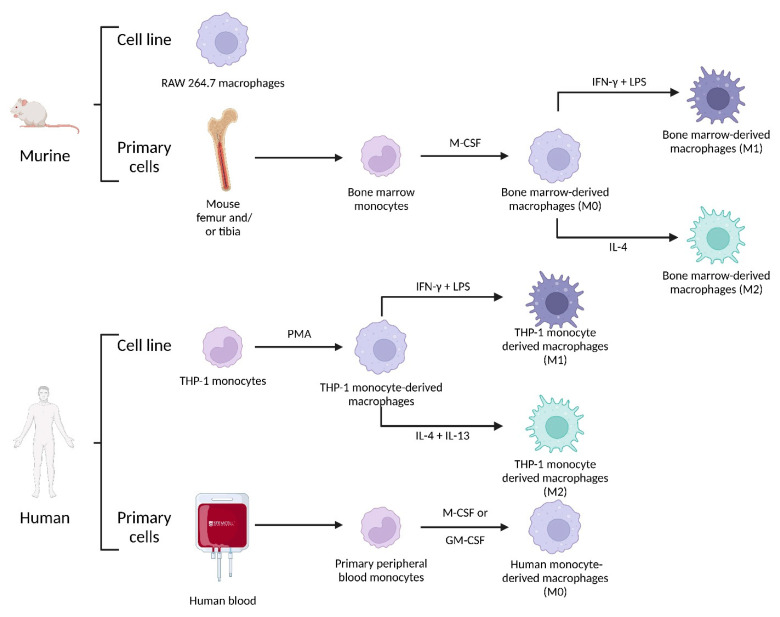
Some of the in vitro models based on murine or human macrophages. Created with BioRender.com.

Since macrophages play an important role in immunosurveillance supported by major phagocytic activity and given their significant presence in tumors, it is essential to assess the impact of SPIONs, as vectors of anti-cancer therapies, on these cells and their precursors (monocytes), whether from safety (cytotoxicity, inflammation) or functional perspectives (polarization, biological responses). In this sense, several recent studies have examined the impact of SPIONs based on in vitro models of monocytes and macrophage cells. All in vitro macrophage models described in this review that were used to study SPIONs are detailed in Figure 4. These macrophage models can also be found in the first two columns of Table 3, a table that depicts the effects of SPIONs on monocyte/macrophage polarization and biological responses.

One of the first parameters to take into account when evaluating the impact of SPIONs on monocytes or macrophages is whether these nanoparticles can undergo rapid uptake. In general, monocytes or macrophages are able to uptake SPIONs relatively rapidly (few hours) [114,170]. Wu et al. demonstrated in primary human monocyte cells that SPIONs can be identified in phagosomes or in cytoplasm [171]. However, there are two noteworthy items regarding the cellular uptake of SPIONs: their size and their charge. Indeed, SPIONs with a size up to 150 nm show a high uptake (Table 3), whereas those with a size above 200 nm showed limited cellular uptake [172]. Zhang et al. [114] demonstrated that the surface charge of SPIONs influenced their uptake rate by murine macrophages. Thus, positively charged SPIONs (+) have a higher rate of uptake than negatively charged SPIONs (−), and negatively charged SPIONs (−) in turn displayed a higher uptake rate than neutral SPIONs (N). Sharkey et al. [173] have also demonstrated that positively charged SPIONs (DEAE-Dextran) provided the best uptake when compared to negatively (CM-Dextran) or neutral (Dextran) ones.

Another important parameter to consider is the impact of SPIONs on cell viability in order to take advantage of the beneficial effects provided by anti-cancer therapies while minimizing the harmful adverse effects potentially induced by SPION vectors, especially since SPIONs cytotoxicity remains unclear [171]. In fact, several variables considerably complicate the evaluation of SPION cytotoxicity. These variables include, for instance, the duration of cell exposure to SPIONs. In order to reduce SPION-related cytotoxicity, Sharkey et al. [173] reduced from 24 h or 48 h to 4 h the incubation time of SPIONs with bone marrow derived-macrophages and no significant decrease in cell viability or increase in cytotoxicity was observed.

**Table 3:** Impact of SPION treatment on monocytes and macrophages.

Species	Cell Model	SPIONs	Biological Responses	Polarization Markers	References
Murine	RAW 264.7	SPIONs (+): +44.72 mV Size: 19.4 nm	Important uptake Cytotoxicity	Increase in iNOS and TNF-α Decrease in IL-10 and VEGF Increase in CD80 and decrease in CD206 in vivo	[114]
Murine	RAW 264.7	SPIONs (−): −27.31 mV Size: 21.3 nm	Important uptake Cytotoxicity	Increase in iNOS and TNF-α Decrease in IL-10 and VEGF Decrease in CD206 in vivo	[114]
Murine	RAW 264.7	SPIONs (N): −0.282 mV Size: 15.9 nm	Uptake No cytotoxicity	Increase in TNF-α Decrease in IL-10 and VEGF Slight decrease in CD206 in vivo	[114]
Murine	RAW 264.7	PEI-SPIONs (+): from +52.2 to +67.1 mV Size: 139–144 nm	Activation of TLR4, MAPK (p44/p42; p38; JNK) ROS production Modulation of podosome formation	Increase in CD40, CD80, CD86 and IL-12 Increase in IL10	[174]
Murine	Bone Marrow-Derived Macrophages	DEAE-Dextran 1:4 (+): +16.8 mV Size: 68 nm	Low cell viability reduction Low cytotoxicity High iron uptake No impact on phagocytosis	Increase in CD86, IL-1β, IL-12β and TNF-α Decrease in CD206 and Arg1	[173]
Murine	Bone Marrow-Derived Macrophages	CM-Dextran (−): −11.6 mV Size: 34.3 nm	Reduction in cell viability Low cytotoxicity Low iron uptake	NR	[173]
Murine	Bone Marrow-Derived Macrophages	Dextran (N): −3.3 mV Size: 36 nm	Reduction in cell viability Low cytotoxicity Low iron uptake	NR	[173]
Murine	Bone Marrow-Derived Macrophages	Resovist^®^: Ferucarbotran Carboxydextrane-coated SPIONs Size: 45–60 nm; core size: 5.8 nm	Activation of TLR4	Increase in IL-1β, IL-2, IL-12, CCL2 and TNF-α	[175]
Murine	Bone Marrow-Derived Macrophages	DMSA SPIONs (−): −29.3 mV Size: 65 nm; core size: 10 nm	Fast uptake No reduction in cell viability Activation of MAPK (ERK) and AKT Decrease in transferrin receptor ROS production	Increase in IL-23α and CCL2 No variation in IL-12 Increase in IL-10	[176]
Murine	Bone Marrow-Derived Macrophages	APS SPIONs (+): +33.3 Mv Size: 54 nm; core size: 8.3 nm	Fast uptake No reduction in cell viability Activation of MAPK (ERK) and AKT Decrease in transferrin receptor Important ROS production	Increase in IL-23α and CCL2 No variation in IL-12 Increase in IL-10	[176]
Murine	Bone Marrow-Derived Macrophages	AD SPIONs (+): +40.3 nm Size: 150 nm; core size: 6.8 nm	Fast uptake No reduction in cell viability Activation of MAPK (ERK) and AKT Decrease in transferrin receptor Important ROS production	Increase in IL-23α and CCL2 No variation in IL-12 No variation in IL-10	[176]
Human	THP-1 monocytes	Dextran-coated SPIONs Size: 83.5 and 86 nm; core size: 6.48 nm	Fast uptake	No increase in CD14, CD11b or CD86 Increase in IL-1β secretion Slight decrease in IL-10 secretion	[170]
Human	THP-1 Monocyte-derived macrophages	Dextran-coated SPIONs Size: 83.5 and 86 nm; core size: 6.48 nm	Fast uptake	No variation in CD14, CD11b or CD86 No variation in IL-1β No variation in IL-10 secretion	[170]
Human	THP-1 Monocyte-derived macrophages	Resovist^®^: Ferucarbotran Carboxydextrane-coated SPIONs Size: 45–60 nm; core size: 5.8 nm	Increase in Ferritin	Increase in CD86 and TNF-α on M2 macrophages	[177]
Human	THP-1 Monocyte-derived macrophages	DMSA SPIONs (−): −29.3 mV Size: 65 nm; core size: 10 nm	Fast uptake No reduction in cell viability Activation of MAPK (ERK) and AKT No activation of p38 nor JNK Decrease in transferrin receptor ROS production	Increase in CD86, TGF-β No variation in IL-12, IL-23α nor CCL2Increase in IL-10	[176]
Human	THP-1 Monocyte-derived macrophages	APS SPIONs (+): +33.3 Mv Size: 54 nm; core size: 8.3 nm	Fast uptake No reduction in cell viability Activation of MAPK (ERK) and AKT No activation of p38 nor JNK Decrease in transferrin receptor and FPN-1 ROS production	Increase in CD86, TGF-β No variation in IL-12, IL-23α nor CCL2 Increase in IL-10	[176]
Human	THP-1 Monocyte-derived macrophages	AD SPIONs (+): +40.3 nm Size: 150 nm; core size: 6.8 nm	Fast uptake No reduction in cell viability Activation of MAPK (ERK) and AKT No activation of p38 nor JNK Decrease in transferrin receptor and FPN-1 ROS production	No variation in CD86, IL-12, IL-23α nor CCL2	[176]
Human	Primary peripheral blood monocytes	Dextran-coated SPIONs Size: 83.5 and 86 nm; core size: 6.48 nm	Fast uptake	NR	[170]
Human	Primary peripheral blood monocytes	Starch-coated SPIONs (−) Size: 200 nm	Low uptake No toxic effects Disruption of actin skeleton	Decrease in IL-6 No variation in IL-1β No variation in IL-10	[172]
Human	Primary peripheral blood monocytes	Dextran SPIONs (−): −11 mV Size: 62,8 nm	Uptake in phagosomes or cytoplasm No decrease in cell viability nor cytotoxicity Activation of MAPK (ERK; p38; JNK)	Increase in IL-1β and TNF-α	[171]
Human	Human Monocyte-derived macrophages	DEAE-Dextran 1:4 (+): +16.8 mV Size: 68 nm	Important cell viability reduction Cytotoxicity Iron uptake	NR	[173]
Human	Human Monocyte-derived macrophages	Resovist^®^: Ferucarbotran Carboxydextrane-coated SPIONs Size: 45 and 60 nm; core size: 5.8 nm	Increase in Ferritin	NR	[177]

Abbreviations: AD: AminoDextran; APS: 3-AminoPropyl-triethoxySilane; DEAE: DiEthylAminoEthyl; DMSA: DiMercaptoSuccinic Acid; ERK: Extracellular signal-Regulated Kinase; FPN-1: FerroPortiN-1; JNK: Jun N-terminal Kinase; MAPK: Mitogen-Activated Protein Kinase; NR: Not Reported; N: Neutral; PEI: Polyethylenimine; ROS: Reactive Oxygen Species; TLR-4: Toll-Like Receptor 4. Additional descriptions: Effects described concern in vitro models unless otherwise specified. The mV number concerns the Z potential. Cellular uptake assays (e.g., iron assays) were performed to determine whether SPIONs are internalized in cells, while cytotoxicity assays (e.g., ATP assays) attempted to evaluate the degree of SPION toxicity.

In this particular case, Sharkey et al. aimed at labeling macrophages with SPIONs before injecting them in mice in order to visualize SPIONs-labelled macrophages by MRI. Therefore, reducing incubation time for ex vivo labeling is possible (for imaging purposes) [173]. However, for studies that aim at evaluating treatments with SPIONs (therapy purposes), systemic administration of SPIONs does not allow the control of the incubation time. In order to reduce cytotoxicity, experiments have shown that SPIONs coated with biocompatible polymers such as dextran, polyethylene glycol, or starch were less cytotoxic [178,179]. Most of the SPIONs listed in Table 3 were coated with these molecules. Another means of decreasing cytotoxicity is to choose biocompatible iron oxides cores such as magnetite (Fe_3_O_4_), maghemite (γ-Fe_2_O_3_), or hematite (α-Fe_2_O_3_) [179] and adapt their concentration below 100 mg/mL [170].

**Figure 5:**
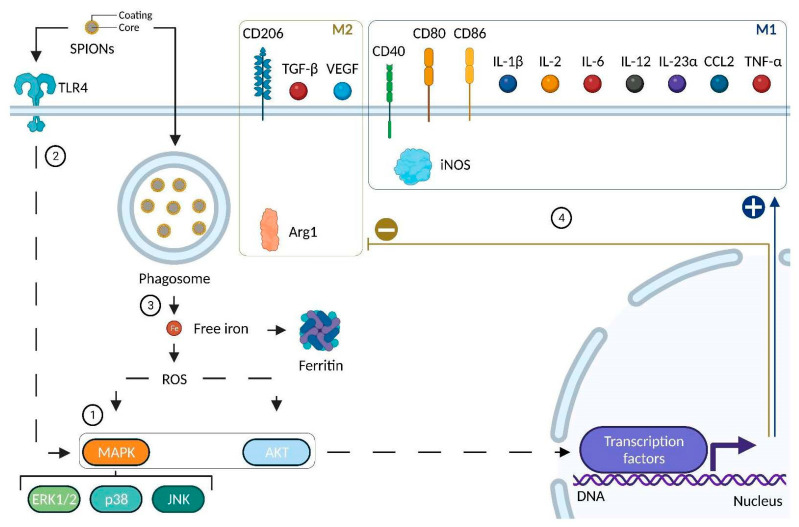
Impact of SPION treatment on polarization markers of macrophages. Created with BioRender.com.

There is no doubt that SPIONs exert an important modulation of macrophages’ biological responses. Kodali et al. showed in a bone-marrow-derived macrophages model that 1052 genes were differently expressed between macrophages treated with SPIONs and controls [180]. The challenge resides in the understanding of which cell signaling pathways are involved. Several studies clearly demonstrated that SPIONs activate the MAPK signaling pathway through the phosphorylation of the downstream mediator ERK1/2 [171,174,176] (Figure 5, 1). One of the most important signaling pathways implied in cell proliferation is the MAP kinase pathway. This signaling pathway can also be activated in case of stress such as DNA damage or heat shock. In this case, the effects of this pathway will be more oriented toward differentiation or apoptosis rather than cell proliferation [181]. Three studies clearly demonstrated that SPION treatment activates the MAPK signaling pathway [171,174,176]. Indeed, downstream mediator ERK1/2 was phosphorylated in those studies. Interestingly, the activation of other MAPK downstream mediators (other than ERK1/2) has been shown to be highly dependent on the type of SPION coating or cellular model used in these studies. As such, PEI-coated SPIONs activated p38 and JNK downstream mediators in RAW 264.7 macrophages [174] as well as dextran-coated SPIONs in primary peripheral blood monocytes [171]. However, DMSA-, APS-, and AD-coated SPIONs induced no phosphorylation of p38 nor JNK in THP-1 cells (monocyte cell line) [176] (Figure 5, 1). Studies have demonstrated that the action of SPIONs on macrophages is, at least in part, mediated by the family of toll-like receptors (TLRs). TLRs are receptors present on cells of the innate immune system, mainly monocytes and macrophages. There are so far ten TLRs that have been discovered in humans [182]. The ligands recognized by these receptors are very variable, either by their structure (LPS, LTA, peptidoglycans, flagellin, RNA, DNA) or by their origin (derived from bacteria, viruses, parasites, or *fungi*) [183]. It has been demonstrated that there is a crosstalk between MAPK and TLR signaling pathways in THP-1 cells, especially with TLR4 [184], the receptor that binds LPS [185] (Figure 5, 2). Moreover, since PEI was linked to the activation of TLR4 [186], Mulens-Arias et al. have demonstrated that the TLR4 signaling pathway is also activated by PEI-coated SPIONs, at least partly, since an inhibitor of TLR4 (CLI-095, also known as TAK-242) reduced IL-1β and VEGFA mRNA induction upon PEI-coated SPION treatment [174]. Jin et al. likewise demonstrated that TLR4 was involved following a SPION treatment [175]. Finally, another signaling pathway that has been described as being activated by SPIONs is the AKT signaling pathway, which could be activated by metabolic stress, such as ROS production [187], which will be discussed below. Indeed, Rojas et al. showed that DMSA-, APS- and AD-coated SPIONs activated the AKT signaling pathway in murine bone marrow-derived macrophages [176] (Figure 5, 1). One point that remains to be elucidated is whether SPIONs activate these various signaling pathways through their interaction with cell membrane receptors (e.g., TLR4) or classical internalization (e.g., phagocytosis), or both. Other signaling pathways should also be studied in detail, such as G protein-coupled receptors, knowing that there is a link between ROS production and AMPK phosphorylation [187], or cytokines and JAK (janus kinases) protein activation, since their triggering has already been shown in an in vitro model of human endothelial cells following a nanoparticle treatment [188].

SPIONs have also been described as directly impacting macrophage iron uptake as well as the expression level of iron-related proteins [173,176,177]. SPIONs are incorporated and degraded inside macrophages. Since the core of SPIONs is composed of iron, their degradation results in an increase in intracellular iron concentration. This iron accumulation in macrophages is thought to promote an M1-like phenotype [189]. M1-like macrophages display an iron storage phenotype. Consequently, these cells express higher levels of proteins involved in iron retention such as ferritin (a multimeric protein that is the main iron storage complex in cells [190]) or transferrin receptor 1 also known as CD71 (a transmembrane protein involved in iron uptake thanks to its binding to iron-loaded transferrin [191]). Conversely, M2-like macrophages present an iron export phenotype with an increase in ferroportin (a transmembrane protein involved in iron release [190]). In this context, Laskar et al. have demonstrated that SPIONs increase the expression of ferritin on THP-1 and human monocyte-derived macrophages [177]. Moreover, SPION treatment caused a decrease in transferrin receptors in M2 bone marrow-derived macrophages as well as in THP-1 monocyte-derived macrophages [176]. Moreover, ferroportin-1 expression was also decreased after 48h following AD- and APS-coated SPION treatment in THP-1 monocyte derived-macrophages [176]. Taken together, these results demonstrate that SPIONs will tend to cause iron accumulation in macrophages, a feature mainly observed in M1-like macrophages.

SPIONs degradation by macrophages may result in free iron atoms in the cytoplasm [192]. These atoms can in turn promote reactive oxygen species (ROS) production in a non-enzymatical way (Fenton chemistry, Figure 5, 3) [193]. In macrophages, ROS are associated with a pro-inflammatory M1-like phenotype since their production is used to destroy pathogens by a mechanism known as respiratory or oxidative burst [194] triggering inflammation [195] via the activation of the NF-κB (nuclear factor-κ B) signaling pathway. PEI-, DMSA-, APS-, and AD-coated SPIONs have been described as inducing ROS production in murine (RAW 264.7 macrophages and bone marrow-derived macrophages) or human (THP-1 monocyte-derived macrophages) macrophages [174,176]. In addition, depending on the type of coating, ROS production levels may vary. AD-coated SPION treatment resulted in more ROS production than SPIONs coated with DMSA in murine macrophages derived from bone marrow [176]. ROS overproduction forms an integral part of oxidative stress that can be deleterious to cells, especially macrophages. The impact of SPIONs on ROS production must be carefully assessed in order to avoid cytotoxic effects linked to oxidative stress and maximize their safety [192].

Other macrophage biological responses have been demonstrated following SPION treatment. DEAE-dextran-coated SPIONs have no impact on macrophage phagocytic activities [173]. This is particularly interesting since one of the main roles of macrophages, phagocytosis, allows them to monitor their microenvironment against possible pathogens and ensure clearance of cellular debris leading to tissue homeostasis [196]. A treatment that would dampen this key feature could therefore prove to be deleterious to the organism. Mulens-Arias et al. demonstrated that PEI-coated SPIONs induced podosome formation in RAW 264.7 macrophages [174], and Gonnissen et al. showed that starch-coated SPIONs led to the disruption of the cytoskeleton in human monocytes [172]. These results all point in the same direction and may suggest that even though SPIONs do not impact the phagocytic activity of macrophages, they could somehow stimulate it indirectly since there is a link between phagocytosis and the formation of podosomes and their transient disruption in human macrophages [197].

Last, but certainly not least, it is also important to highlight that SPIONs exert an impact on macrophage polarization. Different polarization markers (M1 or M2) mainly from three molecule families (enzymes, membrane receptors, and cytokines) vary following SPION treatment (Figure 5, 4).

Firstly, in murine macrophages (RAW 264.7 or bone marrow-derived macrophages), SPIONs increased the expression of iNOS (M1 marker) and decreased the expression of Arg1 (M2 marker) [114,173].

Secondly, the expression of M1-like membrane receptors such as CD40 or CD80 in RAW 264.7 macrophages was increased following SPION treatment [174]. The expression of CD86, another widely used M1 marker, was increased as well when PEI-, DEAE-, Carboxydextrane-, DMSA- and APS-coated SPIONs were used regardless of whether this was assessed on murine or human macrophages [173,174,176,177]. However, no increase in CD86 expression was observed with dextran- or AD-coated SPIONs in human macrophages (THP-1) [170,176]. In addition, a decrease in the expression of M2-like membrane receptor CD206 expression was observed following DEAE-dextran-coated SPION treatment in murine macrophages from bone marrow [173]. An emphasis is needed in one experiment led by Zhang et al. [114]. Murine macrophages (RAW 264.7) were treated with SPIONs and then co-injected with HT1080 cells (fibrosarcoma cell line) in mice. Then, tumors were harvested and analyzed by immunohistochemistry. Compared to the control group (tumor cells co-injected with untreated macrophages), the treated group (tumor cells co-injected with macrophages pre-treated with SPIONs (+)) display an increase in CD80 (M1 marker) and a decrease in CD206 (M2 marker) in vivo. Moreover, SPION (+) pre-treated macrophages were shown to have an important tumor inhibition ability since tumor growth was reduced by at least threefold vs. the control group. These results showed that SPIONs (+) could repolarize macrophages and inhibit tumor growth.

Thirdly, cytokines (interleukins, chemokines, TNF-α, TGF-β, VEGF) expression was evaluated after SPION treatment. In general, SPION treatment had induced an increase in the expression of pro-inflammatory cytokines such as IL-1β, IL-2, IL-6, IL-12, IL-23α, CCL2, and TNF-α in murine or human macrophages [114,170,171,173,174,175,176,177]. Once again, the effects observed may be different for the type of coating and the cell model of use. Thus, in bone marrow-derived macrophages, Carboxydextrane-coated SPIONs induced an increase in IL-12 while DMSA-, APS-, and AD-coated SPIONs did not [175,176]. There is one aspect, however, that must be underlined. Human primary peripheral blood monocytes were treated with dextran-coated SPIONs. This treatment induced the production of pro-inflammatory cytokines such as IL-1β and TNF-α at similar levels to those induced by LPS treatment [171]. This point needs to be further investigated, above all with a view to intravenous treatment with SPIONs. Moreover, the expression of anti-inflammatory cytokines, IL-10 and TGF-β, was also altered by SPION treatment in murine or human macrophages. However, no clear trend was observed. In murine macrophages (RAW 264.7), a decrease and an increase in IL-10 levels has been noted depending on the type of SPIONs being used [114,175]. In human macrophage models, an increase [176], a decrease [170], and no variation [172] in IL-10 levels have been described following SPION treatment. The expression of the other anti-inflammatory cytokine, TGF-β, was increased [176] in THP-1 monocyte-derived macrophages when treated with DMSA- and APS-coated SPIONs. It would be interesting to investigate the variation in this cytokine with different SPIONs in other cell models. Finally, in murine macrophages (RAW 264.7), VEGF expression has been found to decrease following SPION treatment. Since VEGF is considered a marker of M2 polarization involved in angiogenesis [198], it would be worth checking its variation in macrophages from human origin.

To summarize, SPIONs appear to globally induce a trend towards an M1-like pro-inflammatory phenotype (Figure 5) in macrophages (increase in M1- and decrease in M2-associated markers). Despite the fact that cytotoxicity and inflammation related to SPIONs remain issues to be improved, having nanoparticles in the context of cancer biology that would repolarize M2-like macrophages into M1-like macrophages may appear appealing.

In conclusion, given the great heterogeneity of SPIONs (size, surface charge, shape, coating, core composition), it is essential to evaluate the impact of newly synthesized SPIONs on the monocyte-macrophage axis, preferably on primary cell lines as they are closer to physiological conditions and human pathologies [199].

## 5. Conclusions-Perspectives-Outlook

Cancer immunotherapy has tremendous promise, but it has yet to be clinically applied in a wider variety of tumor situations. The main difficulties are toxicity and therapeutic responsiveness limited to a small subset of patients. The variation in patient response rates reflects the various paths tumors use to regulate the various immune-evasion mechanisms occurring in the tumor microenvironment. As a result, it appears that immunotherapy focused against one particular protumoral mechanism is not effective enough at producing a noticeable therapeutic impact. To ensure the creation of novel, efficient cancer treatments, it is extremely desirable to combine therapy approaches that simultaneously target several cancer immuno-evasion systems, albeit this may result in higher toxicity. In this way, immunogenic cell death (ICD)-based strategies have attracted a lot of scientific attention to address the current constraints in treating solid tumors. Indeed, ICD triggers the immune response against the tumor through the activation of dendritic cells, initiating a cascade process leading to an antigen-specific T-cell response. Even though ICD has the effect of boosting the immune system to eliminate the cancer cells, in many instances, the response is insufficient but has been shown to be significantly improved with immune checkpoint inhibitors. ICD can be induced by some chemotherapies (e.g., doxorubicin, 5-fluorouracil) and external beam therapies such as radiotherapy, photodynamic therapy, and hyperthermia.

On that basis, it seems that nanomedicines can offer the possibility of combining these different approaches in the same drug and thus considerably improve the effectiveness of cancer immunotherapy. Indeed, according to their design and the materials they are made of, NPs can act as drug-delivery vehicles and be sensitive to a physical stimulus for either diagnosis and/or therapy (theranostic potential). As vehicles for the precise delivery of tumor antigens and/or immunostimulatory molecules to specific cells located in lymphoid organs or in the tumor microenvironment, nanoparticle-based delivery systems have recently demonstrated a great potential to improve the effectiveness and safety profile of conventional immunotherapeutics. Among these nanomedicines, magnetic NPs such as SPIONs might have enormous potential for safe, more efficient, and individualized cancer treatment. SPIONs have strong biomedical potential because of their high stability, biocompatibility, and low toxicity. Like most nanomedicines, SPIONs enable localized delivery of payload drugs. They also allow us to perform a rational design of novel combinatorial therapies based on immunotherapeutic treatments. In this way, they can target the adaptive and/or innate immune system through their use with/as immunomodulatory therapies (e.g., M2-like TAMs polarization to M1-like phenotype), therapeutic vaccines, and adoptive cell therapies (e.g., cell tracking of chimeric antigen receptor (CAR) T cells). Moreover, and this is what differentiates them from other NPs, due to their distinct ability to react only to an applied external magnetic field, SPIONs are attracting a lot of attention. Indeed, this property is particularly intriguing for biomedical applications and has allowed the development of novel immunotherapeutic approaches that rely on heating capability (magnetic hyperthermia, thermoresponsive drug release), magnetically controlled navigation (i.e., to guide drugs and cell therapies at the target region under a magnetic field), and image-guided techniques, such as magnetic resonance imaging and magnetic particle imaging, a new SPION-based molecular imaging technique. Moreover, due to their versatility, SPIONs make it possible to perform multimodal imaging such as simultaneous PET-MRI, especially for cell tracking. Combining the two imaging modalities may provide at early time points the fast localization and absolute quantification of radiolabeled SPIONs using PET, while MRI gives high-resolution anatomical background information for long-term NP follow-up. This innovative simultaneous approach allows us to overcome the respective limitations of each modality (i.e., resolution for PET and sensitivity for MRI).

As soon as a nanoparticle must be designed, we have to consider that there must be an ad hoc specification, i.e., making this nanoparticle compatible with its further use as a drug or medical device. For an immunotherapeutic approach, due to the complexity of tumor biology, a disease-driven approach should be proposed for the rational design of SPIONs rather than the traditional formulation-driven approach (“one-size-fits-all”). The specification of tumor-targeted SPIONs with immunotherapeutic capabilities will depend on the application, and it is necessary to take into account their multimodal potential, especially for theranostics:

- Good magnetic properties for imaging (MRI and MPI) and hyperthermia (magnetic core > 10 nm).
- Suitable size for passive tumor targeting through EPR effect (typically below 100 nm).
- Surface chemistry: coating to avoid aggregation, conjugations: with targeting moieties if pharmacological selectivity is desired, bifunctional chelating agents for radiolabeling purposes (nuclear imaging, targeted radionuclide therapy), fluorophores, photosensitizers, etc.
- The shape has to be considered since it is recognized as a parameter affecting the immunological response.
- A requirement for standard and optimized zeta potential values: typically, the higher, the better (good stability with absolute zeta values > |30| mV).

In spite of the strong theranostic potential of SPIONs, the limited quantity of SPION-based nanomedicines in clinical trials and on the market demonstrates a number of challenges to be overcome in order to facilitate their translation from the bench to the bedside. The safety of metallic NPs remains a major concern. To evaluate SPION-based nanomedicine biocompatibility and enhance its therapeutic benefits, a detailed investigation of how it interacts with the host tissues is essential. Previous clinical use of SPION formulations that have received FDA/EMA approval has already shown their acceptable safety and biocompatibility, which is unmatched by other metal-based nanoparticle systems. This offers a benefit in using SPIONs as nanomedicines to boost therapeutic results as improvements in cancer immunotherapy are made. Nevertheless, there are still some major regulatory and industrial hurdles to be overcome prior to reaching the market, due to the complex nature of nanomedicine when compared to conventional pharmaceutical products with a single agent. It is also important to consider the impacts of nanoparticles in general, and metallic NPs such as SPIONs in particular, on the environment, society, and ethics to make them acceptable in a biomedical context.

Overall, the unique properties and versatility of SPIONs pave the way for new approaches in the fields of drug delivery and theranostics for cancer immunotherapy, contributing to the personalization of treatments, especially to manage cancers with high unmet medical needs.

## Figures and Tables

**Table 1:** Types of magnetic materials and their response to the external magnetic field.

Type	Spontaneous Magnetization	Description
Diamagnetism	No	Electron magnetic moment compensation. Magnetic interactions within atoms. No exchange magnetic interaction between atoms and molecules. Weakly repelled by magnetic fields.
Paramagnetism 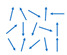	No	Presence of unpaired electrons in the electronic configuration. Weakly attracted by magnetic fields.
Antiferromagnetism 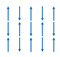	No	Antiparallel ordered magnetic moments. Canting of magnetic moments leading to the appearance of small net magnetization along the direction of the applied magnetic field.
Ferrimagnetism 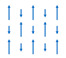	Yes	Antiparallel unbalanced magnetic moments. Small net magnetic moment at zero applied magnetic field.
Ferromagnetism 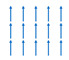	Yes	Parallel magnetic moments. Strong net magnetic moment at zero applied magnetic field.

## Data Availability

Not applicable.
